# Supplementary material for: Class switching toward IgG4 six months after primary mRNA-based COVID-19 vaccination in kidney patients
Source: PLoS One. 2026 Mar 3;21(3):e0336320. doi: 10.1371/journal.pone.0336320 (PMC12956108; doi:10.1371/journal.pone.0336320)
Supplement: S1 Fig — (PDF) [file pone.0336320.s001.pdf]

S1 Fig. Flow cytometry gating strategy

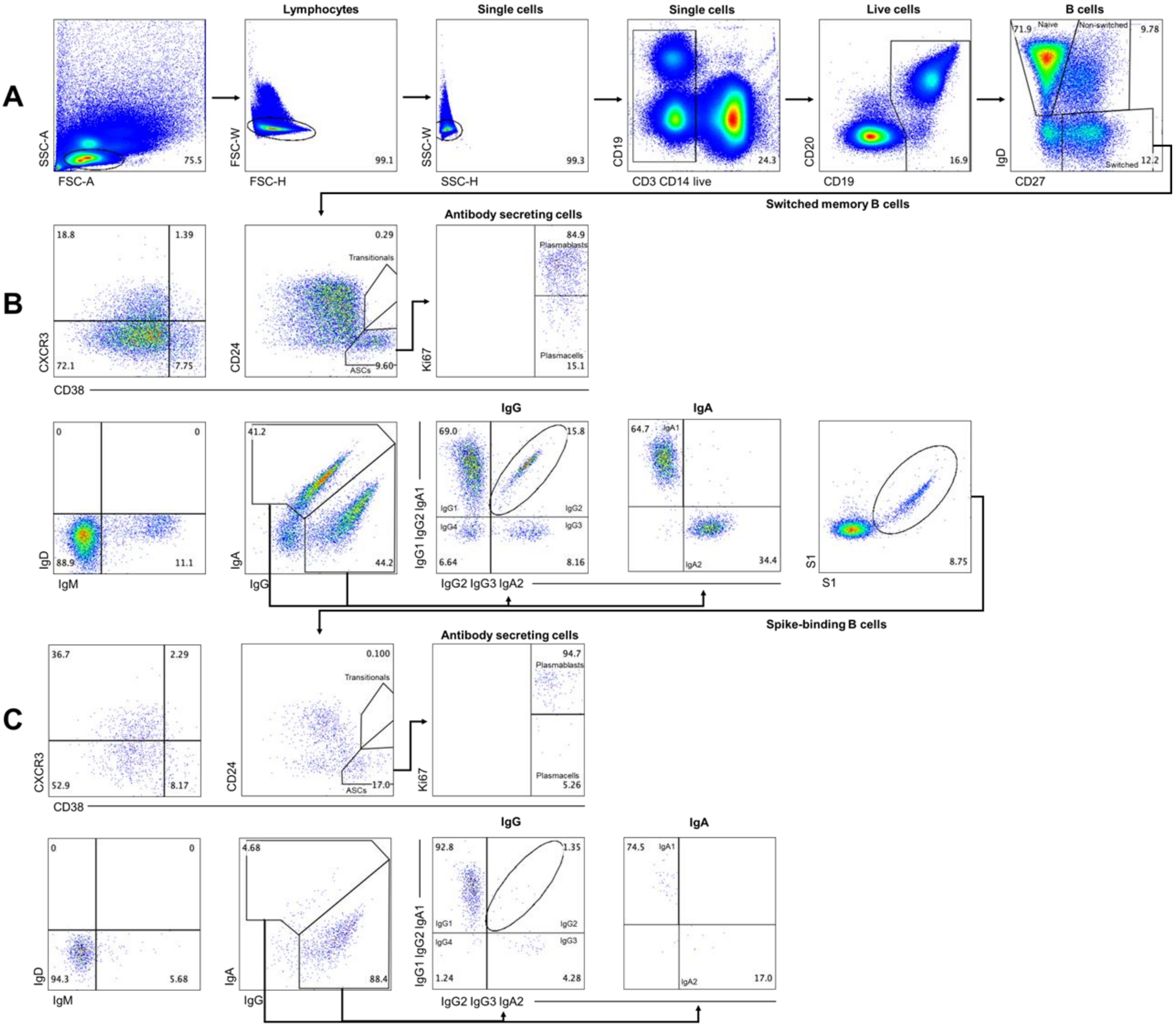

The flow plots are presented from left to right, with the gated populations indicated above each plot.
(A) Lymphocytes were gated, followed by single cells in forward and side scatter, B cells ($CD19^+CD20^+$), and exclusion of T cells (CD3), monocytes (CD14), and dead cells (live), to identify naïve, non-switched, and switched B cells.
(B) Within switched cells, $CXCR3^+$ cells, transitional cells ($CD38^{++}CD24^+$), and antibody-secreting cells (ASCs, $CD38^{++}CD24^-$) were gated; plasma cells ($Ki67^-$) and plasmablasts ($Ki67^+$) were defined within ASCs. IgM^+, IgA^+, and IgG^+ B cells were also identified, and IgG1–3 and IgA1/2 subclasses were distinguished using combinations of PE and FITC with total IgA/IgG antibodies. $IgG4^+$ cells were defined by exclusion within the IgG population (excluding IgA, and subsequently IgG1, IgG2, and IgG3). S-binding B cells were detected by double-positive gating for the two Spike probes.
(C) Within S-binding B cells, the same gating strategy as for switched cells was applied.

Additional information on cell counts: at V3, the number of S-binding B cells ranged from 69 to 690 in CTRLs (average 199, IQR 88–231, out of an average cell input of 2,505,000, IQR 1,560,000–3,087,500), from 15 to 381 in CKD patients (average 42, IQR 24–254, out of 2,270,000, IQR 1,145,000–2,760,000), from 5 to 301 in HD/PD patients (average 30, IQR 8–180, out of 1,410,000, IQR 830,500–1,895,000), and from 30 to 82 in KTRs (average 56, IQR 35–78, out of 2,085,000, IQR 1,562,500–3,237,500). At V4, counts ranged from 69 to 1902 in CTRLs (average 378, IQR 123–546, out of 2,995,000, IQR 1,705,000–3,590,000), from 45 to 1081 in CKD patients (average 189, IQR 79–669, out of 2,620,000, IQR 2,315,000–3,785,000), from 14 to 347 in HD/PD patients (average 38, IQR 20–210, out of 2,000,000, IQR 1,580,000–2,575,000), and from 16 to 260 in KTRs (average 60, IQR 21–176, out of 2,260,000, IQR 1,737,500–3,492,500).
