# Supplementary material for: Class switching toward IgG4 six months after primary mRNA-based COVID-19 vaccination in kidney patients
Source: PLoS One. 2026 Mar 3;21(3):e0336320. doi: 10.1371/journal.pone.0336320 (PMC12956108; doi:10.1371/journal.pone.0336320)
Supplement: S2 Fig — Samples were analyzed at baseline (V1), 28 days post-vaccination (V3), and 6 months post-vaccination (V4). (PDF) [file pone.0336320.s002.pdf]

S2 Fig. Pie chart representation of B cell subsets — naïve (IgD^{+}CD27^{-}), non-switched memory (IgD^{+}CD27^{+}), switched memory (IgD^{-}CD27^{+}), and double negative (IgD^{-}CD27^{-}) — shown as fractions of total CD19+ B cells (100%) over time across all study groups. Samples were analyzed at baseline (V1), 28 days post-vaccination (V3), and 6 months post-vaccination (V4).

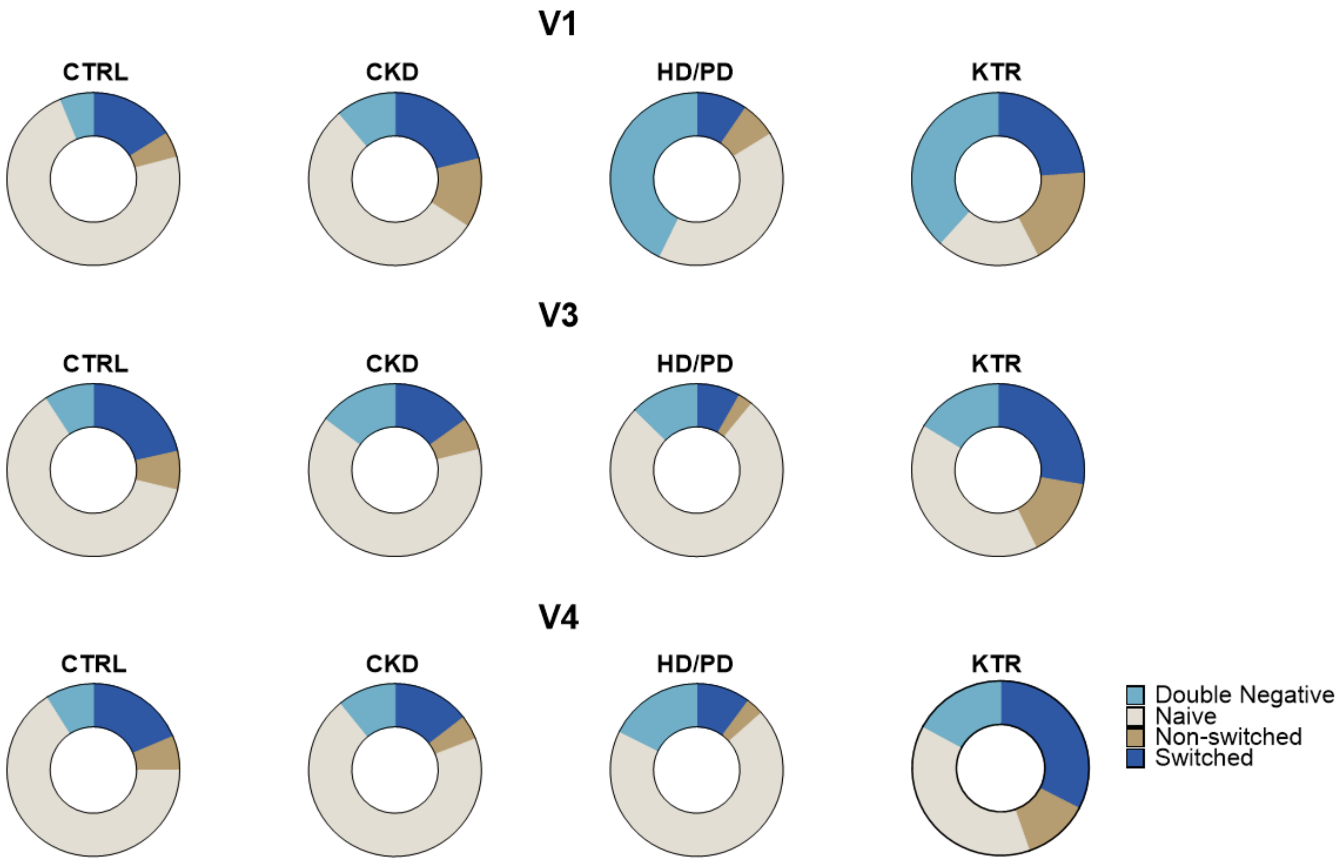

The percentage of switched memory B cells did not significantly change over time within any of the study groups. Median (IQR) values in CTRLs were 17.15 (12.90–34.03) at V1, 20.50 (14.28–31.98) at V3, and 17.75 (13.58–30.13) at V4 (p = 0.935, Kendall's W=0.01). In CKD patients, values were 16.00 (4.10–19.05), 14.90 (4.05–17.60), and 14.30 (3.88–16.60) at V1, V3, and V4, respectively (p = 0.093, Kendall's W=0.52). In HD/PD patients, values were 8.47 (6.42–18.13) at V1, 8.14 (6.59–17.88) at V3, and 8.90 (6.30–17.70) at V4 (p = 0.691, Kendall's W=0.12). In KTRs, values were 26.25 (21.10–32.73), 28.05 (19.23–30.30), and 32.00 (19.60–36.85) at V1, V3, and V4, respectively (p = 0.201, Kendall's W=0.28).

When all participants were analyzed collectively, values were 16.50 (10.10–27.23) at V1, 17.60 (9.91–27.38) at V3, and 16.20 (11.25–29.78) at V4 (p = 0.719, Kendall's W=0.01).
