# Supplementary material for: Class switching toward IgG4 six months after primary mRNA-based COVID-19 vaccination in kidney patients
Source: PLoS One. 2026 Mar 3;21(3):e0336320. doi: 10.1371/journal.pone.0336320 (PMC12956108; doi:10.1371/journal.pone.0336320)
Supplement: S3 Fig — Samples were analyzed at 28 days post-vaccination (V3), and 6 months post-vaccination (V4). (PDF) [file pone.0336320.s003.pdf]

S3 Fig. Pie chart representation of S-binding plasmablasts (Ki67⁺) and plasma cells (Ki67⁻), shown as fractions of antibody-secreting cells (ASCs; 100%) over time in all study groups. Samples were analyzed at 28 days post-vaccination (V3), and 6 months post-vaccination (V4).

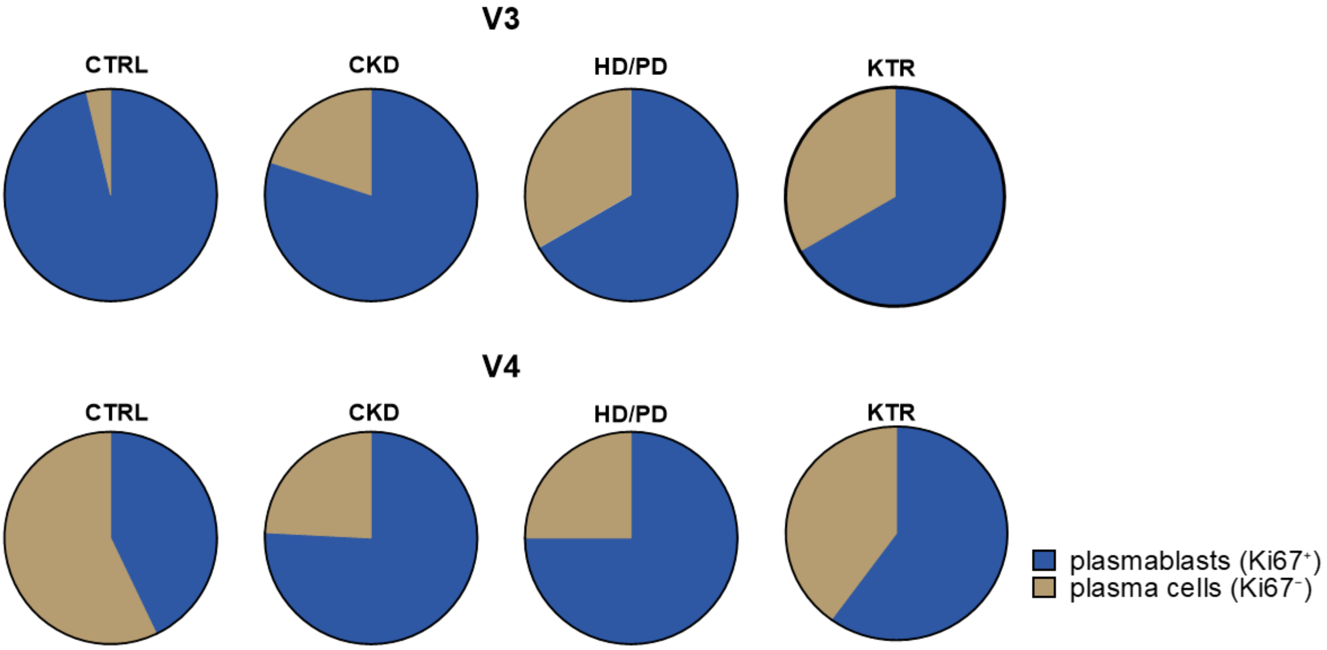

Median (IQR) values in CTRLs were 75.50 (71.25–80.45) at V3 and 50.00 (43.03–53.20) at V4 ($p = 0.016$, $r = -0.85$). In CKD patients, values were 93.30 (78.90–100.0) and 75.80 (61.35–86.45) at V3 and V4, respectively ($p = 0.313$, $r = -0.45$). In HD/PD patients, values were 66.70 (25.00–94.45) at V3 and 85.70 (37.50–93.35) at V4 ($p = 0.875$, $r = 0.07$). In KTRs, values were 66.70 (0.00–86.35) and 71.25 (45.00–100.0) at V3 and V4, respectively ($p = 0.625$, $r = 0.20$). No significant between-group differences were observed at V3 ($p = 0.200$, $\varepsilon^2 = 0.20$) or V4 ($p = 0.156$, $\varepsilon^2 = 0.23$).
