# Supplementary material for: Class switching toward IgG4 six months after primary mRNA-based COVID-19 vaccination in kidney patients
Source: PLoS One. 2026 Mar 3;21(3):e0336320. doi: 10.1371/journal.pone.0336320 (PMC12956108; doi:10.1371/journal.pone.0336320)

S4 Fig. Flow cytometry gating in KTRs with relatively high S-binding IgG4 percentages over time. Samples were analyzed at 28 days post-vaccination (V3, left) and 6 months post-vaccination (V4, right). For each participant, the top row shows total B cells and the bottom row shows S-binding B cells. **(A)** KTR2; **(B)** KTR3; **(C)** KTR4; **(D)** KTR6.

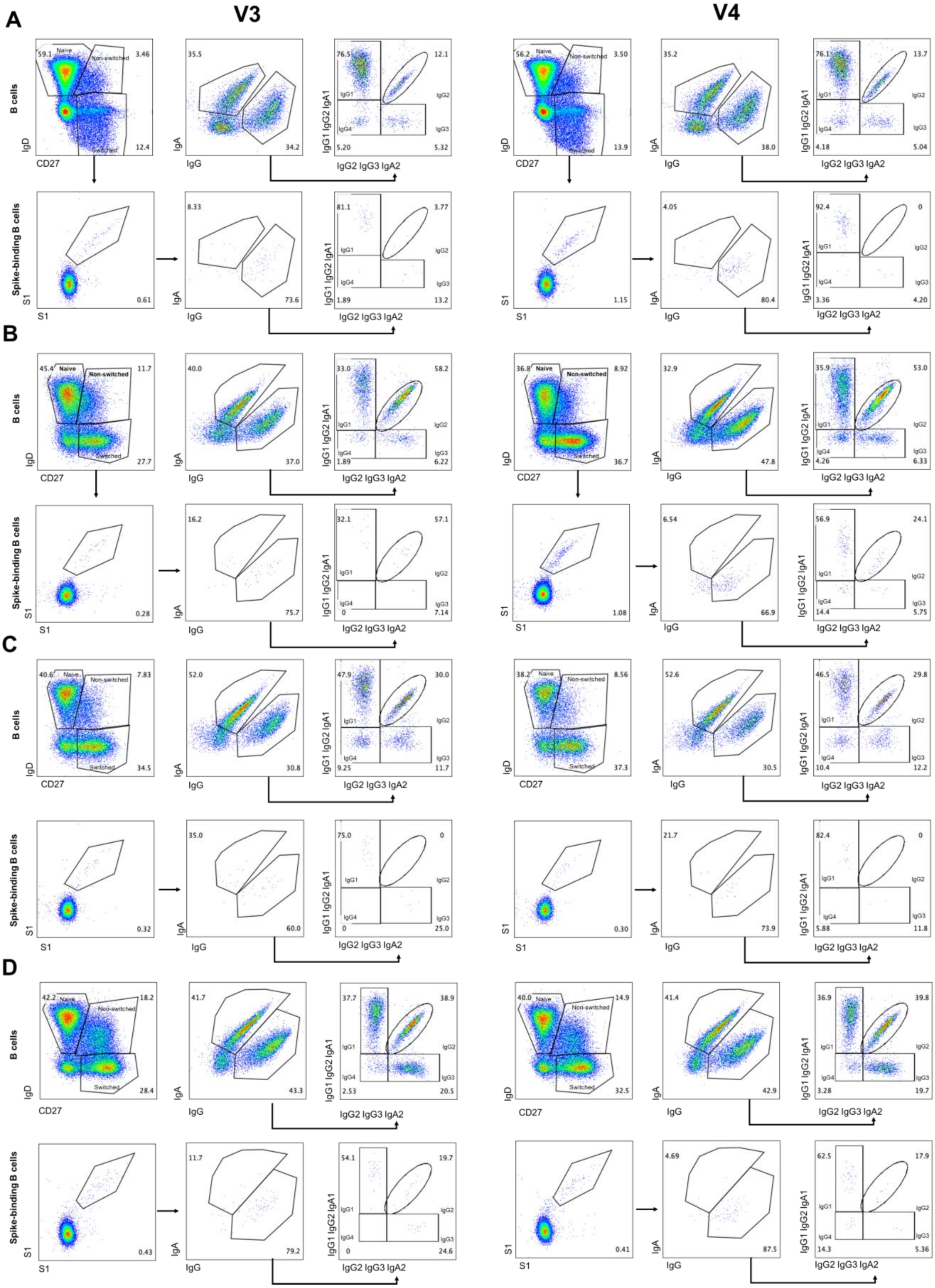

Supplement: S4 Fig — Samples were analyzed at 28 days post-vaccination (V3, left) and 6 months post-vaccination (V4, right). For each participant, the top row shows total B cells and the bottom row shows S-binding B cells. (A) KTR2; (B) KTR3; (C) KTR4; (D) KTR6. (PDF) [file pone.0336320.s004.pdf]
