# Supplementary material for: Class switching toward IgG4 six months after primary mRNA-based COVID-19 vaccination in kidney patients
Source: PLoS One. 2026 Mar 3;21(3):e0336320. doi: 10.1371/journal.pone.0336320 (PMC12956108; doi:10.1371/journal.pone.0336320)
Supplement: S1 Table — (PDF) [file pone.0336320.s005.pdf]

S1 Table. Monoclonal antibodies (mAbs) used in B cell panel.

mAb	Fluorochrome	Clone	Manufacturer	Product number	Surface or intracellular
CD14	BV510	MØPg	BD	563079	surface
CXCR3	R718	1C6/CXCR3	BD	567038	surface
CD38	BUV563	HB7	BD	741446	surface
CD3	BV510	OKT3	BD	566779	surface
CD24	BV650	ML5	BD	563720	surface
CD19	BV785	SJ25C1	BD	563325	surface
CD27	BB700	M-T271	BD	566449	surface
CD20	APC-Fire750	2H7	Biolegend	302358	surface
IgM	BUV395	G20-127	BD	563903	surface
IgD	PE-CF594	IA6-2	BD	562540	surface
IgG	BUV496	G18-145	BD	741172	surface
IgG1	PE	HP6001	Southern Biotech	9054-09	surface
IgG2	PE	HP6002	Southern Biotech	9070-09	surface
IgG2	FITC	HP6002	Southern Biotech	9070-02	surface
IgG3	FITC	HP6050	Southern Biotech	9210-02	surface
IgA	PE-Vio770	REA1014	Miltenyi	130-116-883	surface
IgA1	PE	B3506B4	Abcam	ab99797	surface
IgA2	FITC	IS11-21E11	Miltenyi	130-093-069	surface
Ki67	BV711	Ki-67	Biolegend	350516	intracellular
