# Supplementary material for: Class switching toward IgG4 six months after primary mRNA-based COVID-19 vaccination in kidney patients
Source: PLoS One. 2026 Mar 3;21(3):e0336320. doi: 10.1371/journal.pone.0336320 (PMC12956108; doi:10.1371/journal.pone.0336320)
Supplement: S2 Table — White bars represent included KTRs, light grey bars indicate excluded KTRs with negligible S-binding B-cell counts, and dark grey bars indicate individuals without antibody response who served as negative controls. (PDF) [file pone.0336320.s006.pdf]

S2 Table. Immunosuppressive agents received per patient in the KTR group, including excluded participants. White bars represent included KTRs, light grey bars indicate excluded KTRs with negligible S-binding B-cell counts, and dark grey bars indicate individuals without antibody response who served as negative controls.

	Steroids	Azathioprine	MMF	Calcineurin inhibitor	mTOR inhibitor
**KTR1**	Yes	No	No	Yes	No
**KTR2**	Yes	No	Yes	Yes	No
**KTR3**	Yes	No	No	Yes	No
**KTR4**	Yes	No	No	Yes	No
**KTR5**	Yes	Yes	No	Yes	No
**KTR6**	Yes	No	Yes	Yes	No
**KTR7**	Yes	No	No	Yes	No
**KTR8**	Yes	No	Yes	Yes	No
**KTR9**	Yes	No	No	Yes	No
**KTR10**	Yes	No	No	Yes	No
**KTR11**	Yes	No	No	Yes	No
**KTR12**	Yes	No	Yes	Yes	No
**KTR13**	Yes	No	Yes	Yes	No
**KTR14**	Yes	No	Yes	Yes	No
**KTR15**	Yes	No	Yes	No	No
**KTR16**	Yes	No	Yes	No	Yes
**KTR17**	Yes	No	No	Yes	No
