# Supplementary material for: Class switching toward IgG4 six months after primary mRNA-based COVID-19 vaccination in kidney patients
Source: PLoS One. 2026 Mar 3;21(3):e0336320. doi: 10.1371/journal.pone.0336320 (PMC12956108; doi:10.1371/journal.pone.0336320)
Supplement: S3 Table — (PDF) [file pone.0336320.s007.pdf]

S3 Table. Medians, IQRs, and full statistical comparisons of S1 IgG antibody levels and S-binding B cell frequencies over time.

Group	Timepoint/Population	Median (IQR)	p (vs previous)	Effect size (r/ε²)
CTRL	V3 S1 IgG levels	2194.0 (932.9–4409.0)	—	—
CTRL	V4 S1 IgG levels	310.0 (179.1–530.4)	0.008 (vs V3)	–0.89
CKD	V3 S1 IgG levels	3239.0 (2467.0–6012.0)	—	—
CKD	V4 S1 IgG levels	297.2 (207.4–759.6)	0.063 (vs V3)	–0.90
HD/PD	V3 S1 IgG levels	1418.0 (743.1–2583.0)	—	—
HD/PD	V4 S1 IgG levels	276.4 (24.0–509.2)	0.063 (vs V3)	–0.90
KTR	V3 S1 IgG levels	385.5 (196.7–1252.0)	—	—
KTR	V4 S1 IgG levels	106.6 (55.0–818.1)	0.438 (vs V3)	–0.38
CTRL	V1 S-binding (%)	0.07 (0.03–0.13)	—	—
CTRL	V3 S-binding (%)	0.56 (0.35–0.84)	0.073 (vs V1)	0.56
CTRL	V4 S-binding (%)	0.95 (0.53–2.07)	0.001 (vs V1)	0.94
CKD	V1 S-binding (%)	0.09 (0.06–0.15)	—	—
CKD	V3 S-binding (%)	0.73 (0.39–1.01)	0.342 (vs V1)	0.50
CKD	V4 S-binding (%)	1.34 (0.69–1.96)	0.005 (vs V1)	1.00
HD/PD	V1 S-binding (%)	0.06 (0.02–0.13)	—	—
HD/PD	V3 S-binding (%)	0.56 (0.20–1.02)	0.081 (vs V1)	0.70
HD/PD	V4 S-binding (%)	0.82 (0.27–1.21)	0.034 (vs V1)	0.80
KTR	V1 S-binding (%)	0.10 (0.06–0.20)	—	—
KTR	V3 S-binding (%)	0.38 (0.31–0.54)	0.028 (vs V1)	0.75
KTR	V4 S-binding (%)	0.36 (0.23–1.10)	0.250 (vs V1)	0.50
