# Supplementary material for: Class switching toward IgG4 six months after primary mRNA-based COVID-19 vaccination in kidney patients
Source: PLoS One. 2026 Mar 3;21(3):e0336320. doi: 10.1371/journal.pone.0336320 (PMC12956108; doi:10.1371/journal.pone.0336320)
Supplement: S4 Table — (PDF) [file pone.0336320.s008.pdf]

S4 Table. Medians, IQRs, and full statistical comparisons of circulating ASCs and CXCR3$^+$ B cells within S-binding and switched memory B cell populations.

Group	Timepoint/Population (%)	Median (IQR)	p	Effect size (r/ε²)
CTRL	V3 ASCs (switched)	1.66 (0.82–4.09)	—	—
CTRL	V4 ASCs (switched)	2.68 (0.85–4.92)	—	—
CKD	V3 ASCs (switched)	3.86 (2.62–5.14)	—	—
CKD	V4 ASCs (switched)	4.20 (1.95–7.24)	—	—
HD/PD	V3 ASCs (switched)	3.31 (1.98–9.32)	—	—
HD/PD	V4 ASCs (switched)	2.46 (1.75–13.78)	—	—
KTR	V3 ASCs (switched)	7.23 (2.18–11.18)	—	—
KTR	V4 ASCs (switched)	7.14 (1.96–10.39)	—	—
CTRL	V3 ASCs (S-binding)	10.37 (5.95–14.05)	vs switched: 0.008	0.89
CTRL	V4 ASCs (S-binding)	7.74 (5.39–11.37)	vs switched: 0.008<br>vs V3: 0.461	0.89<br>-0.30
CKD	V3 ASCs (S-binding)	11.90 (6.37–17.25)	vs switched: 0.063	0.90
CKD	V4 ASCs (S-binding)	8.89 (5.70–12.60)	vs switched: >0.999<br>vs V3: 0.438	0.78<br>-0.42
HD/PD	V3 ASCs (S-binding)	6.67 (4.04–34.55)	vs switched: 0.438	0.42
HD/PD	V4 ASCs (S-binding)	7.14 (2.16–10.11)	vs switched: 0.813<br>vs V3: 0.813	0.18<br>0.18
KTR	V3 ASCs (S-binding)	3.20 (1.83–11.33)	vs switched: >0.999	-0.81
KTR	V4 ASCs (S-binding)	3.74 (2.31–12.87)	vs switched: >0.999<br>vs V3: 0.844	-0.81<br>0.64
CTRL	V3 CXCR3 (switched)	1.54 (0.615–8.03)	—	—
CTRL	V4 CXCR3 (switched)	1.80 (0.53–8.14)	—	—
CKD	V3 CXCR3 (switched)	0.57 (0.38–1.36)	—	—
CKD	V4 CXCR3 (switched)	0.34 (0.26–0.99)	—	—
HD/PD	V3 CXCR3 (switched)	0.61 (0.19–0.97)	—	—
HD/PD	V4 CXCR3 (switched)	0.47 (0.35–0.78)	—	—
KTR	V3 CXCR3 (switched)	5.55 (0.25–6.89)	—	—
KTR	V4 CXCR3 (switched)	5.84 (0.32–8.66)	—	—
CTRL	V3 CXCR3 (S-binding)	11.0 (5.34–35.83)	vs switched: 0.008	0.94
CTRL	V4 CXCR3 (S-binding)	2.46 (0.41–11.0)	vs switched: 0.313<br>vs V3: 0.016	0.36<br>-0.84
CKD	V3 CXCR3 (S-binding)	0.26 (0.0–5.16)	vs switched: 0.813	-0.11
CKD	V4 CXCR3 (S-binding)	0.0 (0.0–2.11)	vs switched: 0.813<br>vs V3: 0.3750	-0.11<br>-0.66
HD/PD	V3 CXCR3 (S-binding)	9.09 (0.0–12.3)	vs switched: 0.313	0.45

HD/PD	V4 CXCR3 (S-binding)	0.0 (0.0–2.07)	vs switched: 0.625<br>vs V3: 0.250	-0.22<br>-0.90
KTR	V3 CXCR3 (S-binding)	20.45 (2.50–48.43)	vs switched: 0.063	0.76
KTR	V4 CXCR3 (S-binding)	14.05 (0.51–22.13)	vs switched: 0.063<br>vs V3: 0.156	0.76<br>-0.64
