# Supplementary material for: Class switching toward IgG4 six months after primary mRNA-based COVID-19 vaccination in kidney patients
Source: PLoS One. 2026 Mar 3;21(3):e0336320. doi: 10.1371/journal.pone.0336320 (PMC12956108; doi:10.1371/journal.pone.0336320)
Supplement: S5 Table — (PDF) [file pone.0336320.s009.pdf]

S5 Table. Medians, IQRs, and full statistical comparisons of frequencies of IgM$^+$, IgA$^+$, and IgG$^+$ B cells within S-binding and switched memory B cell populations.

Group comparisons were performed using Wilcoxon signed-rank and Kruskal–Wallis tests; effect sizes are reported as r or ε^2.

<u>IgM+ B cells</u>

Group	Timepoint / Population (%)	Median (IQR)	p	Effect size (r / ε^2)
CTRL	V3 (switched)	15.5 (9.8–20.6)	–	–
CTRL	V4 (switched)	16.0 (11.3–27.0)	–	–
CTRL	V3 (S-binding)	8.2 (5.2–25.0)	vs switched V3: p=0.148	r=-0.51
CTRL	V4 (S-binding)	4.9 (3.0–12.6)	vs switched V4: p=0.008<br>vs V3: 0.109	r=-0.94<br>r=-0.57
CKD	V3 (switched)	16.6 (9.7–37.7)	–	–
CKD	V4 (switched)	24.0 (10.8–41.0)	–	–
CKD	V3 (S-binding)	7.1 (2.9–51.0)	vs switched V3: p>0.999	r=0.00
CKD	V4 (S-binding)	12.4 (5.9–23.8)	vs switched V4: p=0.063<br>vs V3: 0.625	r=-0.84<br>r=0.22
HD/PD	V3 (switched)	16.1 (8.9–19.7)	–	–
HD/PD	V4 (switched)	16.6 (12.4–25.1)	–	–
HD/PD	V3 (S-binding)	2.3 (0.0–9.3)	vs switched V3: p=0.063	r=-0.84
HD/PD	V4 (S-binding)	5.6 (3.2–18.4)	vs switched V4: p=0.063<br>vs V3: 0.063	r=-0.84<br>r=0.84
KTR	V3 (switched)	15.2 (9.3–22.4)	–	–
KTR	V4 (switched)	16.5 (8.4–22.2)	–	–
KTR	V3 (S-binding)	10.3 (6.5–29.1)	vs switched V3: p=0.844	r=-0.08
KTR	V4 (S-binding)	10.6 (7.1–30.2)	vs switched V4: p>0.999<br>vs V3: 0.844	r=0.00<br>r=0.08

<u>IgA+ B cells</u>

Group	Timepoint / Population (%)	Median (IQR)	p	Effect size (r / ε^2)
CTRL	V3 (switched)	32.7 (28.0–46.4)	–	–
CTRL	V4 (switched)	32.7 (27.7–45.8)	–	–
CTRL	V3 (S-binding)	12.0 (5.5–15.6)	vs switched V3: p=0.250	r=-0.41
CTRL	V4 (S-binding)	5.7 (2.1–9.0)	vs switched V4: p=0.008<br>vs V3: 0.148	r=-0.94<br>r=-0.51
CKD	V3 (switched)	29.2 (23.6–39.1)	–	–
CKD	V4 (switched)	31.1 (27.8–41.2)	–	–
CKD	V3 (S-binding)	6.7 (2.0–9.5)	vs switched V3: p=0.063	r=-0.84
CKD	V4 (S-binding)	4.3 (2.1–5.3)	vs switched V4: p=0.063<br>vs V3: 0.313	r=-0.84<br>r=-0.45

HD/PD	V3 (switched)	43.2 (33.9–48.5)	–	–
HD/PD	V4 (switched)	41.2 (31.6–47.6)	–	–
HD/PD	V3 (S-binding)	22.0 (7.0–45.0)	vs switched V3: p=0.813	r=-0.11
HD/PD	V4 (S-binding)	2.6 (0.9–11.0)	vs switched V4: p=0.063<br>vs V3: 0.063	r=-0.84<br>r=-0.84
KTR	V3 (switched)	40.9 (35.1–46.6)	–	–
KTR	V4 (switched)	38.3 (34.5–51.9)	–	–
KTR	V3 (S-binding)	10.9 (9.4–20.9)	vs switched V3: p=0.688	r=-0.16
KTR	V4 (S-binding)	6.4 (4.5–18.9)	vs switched V4: p=0.313<br>vs V3: 0.219	r=-0.41<br>r=-0.50

<u>IgG+ B cells</u>

**Group**	**Timepoint / Population (%)**	**Median (IQR)**	**p**	**Effect size (r / ε²)**
CTRL	V3 (switched)	40.4 (33.8–49.0)	–	–
CTRL	V4 (switched)	41.9 (35.4–50.2)	–	–
CTRL	V3 (S-binding)	71.6 (59.4–74.0)	vs switched V3: p=0.008	r=0.94
CTRL	V4 (S-binding)	77.9 (59.0–78.3)	vs switched V4: p=0.008<br>vs V3: 0.195	r=0.94<br>r=0.46
CKD	V3 (switched)	37.1 (25.0–48.2)	–	–
CKD	V4 (switched)	40.1 (27.7–45.4)	–	–
CKD	V3 (S-binding)	66.7 (33.8–79.1)	vs switched V3: p=0.125	r=0.68
CKD	V4 (S-binding)	66.6 (62.8–76.4)	vs switched V4: p=0.063<br>vs V3: 0.813	r=0.84<br>r=0.11
HD/PD	V3 (switched)	29.8 (21.1–45.4)	–	–
HD/PD	V4 (switched)	27.4 (21.5–46.0)	–	–
HD/PD	V3 (S-binding)	63.6 (56.7–74.3)	vs switched V3: p=0.063	r=0.84
HD/PD	V4 (S-binding)	60.5 (47.6–88.7)	vs switched V4: p=0.063<br>vs V3: 0.438	r=0.84<br>r=-0.35
KTR	V3 (switched)	32.5 (29.5–38.6)	–	–
KTR	V4 (switched)	34.3 (29.3–44.1)	–	–
KTR	V3 (S-binding)	74.7 (56.7–79.5)	vs switched V3: p=0.031	r=0.88
KTR	V4 (S-binding)	70.4 (57.4–82.2)	vs switched V4: p=0.031<br>vs V3: 0.563	r=0.88<br>r=-0.24
