# Supplementary material for: Class switching toward IgG4 six months after primary mRNA-based COVID-19 vaccination in kidney patients
Source: PLoS One. 2026 Mar 3;21(3):e0336320. doi: 10.1371/journal.pone.0336320 (PMC12956108; doi:10.1371/journal.pone.0336320)
Supplement: S6 Table — (PDF) [file pone.0336320.s010.pdf]

S6 Table. Medians, IQRs, and full statistical comparisons of frequencies of IgA^+ and IgG^+ S-binding B cell subclasses and correlations of IgG4 with other IgG subclasses.

Group comparisons were performed using Wilcoxon signed-rank and Kruskal–Wallis tests; effect sizes are reported as r or ε^2.

Timepoint / Population (%)	Median (IQR)	p	Effect size (r / ε^2)
IgA1 V3	81.65 (56.65–93.30)	–	–
IgA1 V4	71.00 (45.35–99.23)	p=0.468	r=-0.15
IgA2 V3	1.73 (0.00–15.30)	–	–
IgA2 V4	4.17 (0.00–17.60)	p=0.945	r=0.01
Comparison IgA1 vs IgA2 V3	–	p=0.0002	r=0.76
Comparison IgA1 vs IgA2 V4	–	p<0.0001	r=0.82
IgG1 V3	80.95 (67.53–88.10)	–	–
IgG2 V3	5.60 (2.20–7.55)	–	–
IgG3 V3	9.14 (3.19–13.00)	–	–
IgG4 V3	0.00 (0.00–1.96)	–	–
IgG1 vs IgG2 V3	–	p<0.0001	r=0.79
IgG1 vs IgG3 V3	–	p<0.0001	r=0.65
IgG1 vs IgG4 V3	–	p<0.0001	r=1.00
IgG2 vs IgG3 V3	–	p>0.999	r=-0.14
IgG2 vs IgG4 V3	–	p=0.125	r=0.33
IgG3 vs IgG4 V3	–	p=0.007	r=0.47
IgG1 V4	80.75 (69.78–86.65)	p=0.861 (vs V3)	r=-0.04
IgG2 V4	1.50 (0.00–7.45)	p=0.022 (vs V3)	r=-0.47
IgG3 V4	4.28 (1.70–7.31)	p=0.001 (vs V3)	r=-0.66
IgG4 V4	10.70 (0.37–17.90)	p<0.0001 (vs V3)	r=0.80
IgG1 vs IgG2 V4	–	p<0.0001	r=0.99
IgG1 vs IgG3 V4	–	p<0.0001	r=0.85
IgG1 vs IgG4 V4	–	p<0.0001	r=0.72
IgG2 vs IgG3 V4	–	p>0.999	r=-0.15
IgG2 vs IgG4 V4	–	p=0.321	r=-0.28
IgG3 vs IgG4 V4	–	p>0.999	r=-0.13
