# Supplementary material for: Class switching toward IgG4 six months after primary mRNA-based COVID-19 vaccination in kidney patients
Source: PLoS One. 2026 Mar 3;21(3):e0336320. doi: 10.1371/journal.pone.0336320 (PMC12956108; doi:10.1371/journal.pone.0336320)
Supplement: S7 Table — (PDF) [file pone.0336320.s011.pdf]

S7 Table. Medians, IQRs, and full statistical comparisons of heatmaps showing IgA and IgG subclasses (total 100%) within S-binding and switched memory B cell populations.

Group comparisons were performed using Wilcoxon signed-rank and Kruskal–Wallis tests; effect sizes are reported as r or ε².

IgA1+

Group	Timepoint / Population (%)	Median (IQR)	p	Effect size (r / ε²)
CTRL	IgA1 V3 (switched)	68.80 (53.15–77.20)	–	–
CTRL	IgA1 V4 (switched)	68.85 (54.33–78.25)	–	–
CTRL	IgA1 V3 (S-binding)	74.15 (56.65–86.35)	vs switched V3: p=0.195	r=0.46
CTRL	IgA1 V4 (S-binding)	84.05 (49.03–99.23)	vs switched V4: p=0.055<br>vs V3: 0.297	r=0.68<br>r=0.39
CKD	IgA1 V3 (switched)	66.90 (62.00–80.00)	–	–
CKD	IgA1 V4 (switched)	68.70 (62.80–78.75)	–	–
CKD	IgA1 V3 (S-binding)	93.30 (40.00–100.00)	vs switched V3: p=0.625	r=0.22
CKD	IgA1 V4 (S-binding)	71.40 (35.30–90.90)	vs switched V4: p=0.625<br>vs V3: 0.625	r=0.22<br>r=-0.25
HD/PD	IgA1 V3 (switched)	60.40 (50.25–67.75)	–	–
HD/PD	IgA1 V4 (switched)	64.80 (53.25–72.45)	–	–
HD/PD	IgA1 V3 (S-binding)	92.30 (22.20–96.65)	vs switched V3: p=0.813	r=0.11
HD/PD	IgA1 V4 (S-binding)	40.00 (0.00–90.00)	vs switched V4: p=0.438<br>vs V3: 0.375	r=-0.35<br>r=-0.45
KTR	IgA1 V3 (switched)	66.85 (59.80–68.05)	–	–
KTR	IgA1 V4 (switched)	67.20 (59.68–68.40)	–	–
KTR	IgA1 V3 (S-binding)	79.15 (61.13–94.68)	vs switched V3: p=0.219	r=0.50
KTR	IgA1 V4 (S-binding)	59.40 (50.00–100.00)	vs switched V4: p>0.999<br>vs V3: 0.906	r=0.00<br>r=-0.05
	Between groups IgA1 V3	–	p=0.769	ε²=0.05
	Between groups IgA1 V4	–	p=0.605	ε²=0.08

IgA2+

Group	Timepoint / Population (%)	Median (IQR)	p	Effect size (r / ε²)
CTRL	IgA2 V3 (switched)	29.55 (20.43–42.90)	–	–
CTRL	IgA2 V4 (switched)	29.45 (19.60–42.55)	–	–
CTRL	IgA2 V3 (S-binding)	8.39 (4.26–18.43)	vs switched V3: p=0.016	r=-0.86
CTRL	IgA2 V4 (S-binding)	9.72 (0.00–17.38)	vs switched V4: p=0.008<br>vs V3: 0.563	r=-0.94<br>r=0.21
CKD	IgA2 V3 (switched)	25.70 (18.20–34.70)	–	–

CKD	IgA2 V4 (switched)	25.80 (19.45–34.55)	–	–
CKD	IgA2 V3 (S-binding)	0.00 (0.00–0.00)	vs switched V3: p=0.063	r=-0.84
CKD	IgA2 V4 (S-binding)	9.09 (0.00–26.05)	vs switched V4: p=0.063<br>vs V3: p=0.250	r=-0.84<br>r=0.51
HD/PD	IgA2 V3 (switched)	37.00 (30.10–44.70)	–	–
HD/PD	IgA2 V4 (switched)	32.20 (25.00–43.40)	–	–
HD/PD	IgA2 V3 (S-binding)	6.67 (0.00–72.20)	vs switched V3: p=0.813	r=-0.11
HD/PD	IgA2 V4 (S-binding)	0.00 (0.00–10.00)	vs switched V4: p=0.063<br>vs V3: 0.250	r=-0.84<br>r=-0.51
KTR	IgA2 V3 (switched)	29.65 (28.00–38.03)	–	–
KTR	IgA2 V4 (switched)	30.10 (29.08–37.85)	–	–
KTR	IgA2 V3 (S-binding)	0.00 (0.00–12.50)	vs switched V3: p=0.031	r=-0.88
KTR	IgA2 V4 (S-binding)	8.35 (0.00–18.20)	vs switched V4: p=0.031<br>vs V3: 0.500	r=-0.88<br>r=0.27
	Between groups IgA2 V3	–	p=0.050	ε^2=0.34
	Between groups IgA2 V4	–	p=0.636	ε^2=0.07

IgG1+

**Group**	**Timepoint / Population (%)**	**Median (IQR)**	**p**	**Effect size (r / ε^2)**
CTRL	IgG1 V3 (switched)	57.60 (51.10–60.10)	–	–
CTRL	IgG1 V4 (switched)	58.55 (55.30–63.83)	–	–
CTRL	IgG1 V3 (S-binding)	81.05 (71.40–87.88)	vs switched V3: p=0.016	r=0.86
CTRL	IgG1 V4 (S-binding)	82.65 (70.35–87.98)	vs switched V4: p=0.039<br>vs V3: 0.742	r=0.73<br>r=0.12
CKD	IgG1 V3 (switched)	49.40 (46.25–66.45)	–	–
CKD	IgG1 V4 (switched)	51.10 (49.50–65.15)	–	–
CKD	IgG1 V3 (S-binding)	82.00 (73.75–86.55)	vs switched V3: p=0.063	r=0.83
CKD	IgG1 V4 (S-binding)	78.40 (75.35–84.85)	vs switched V4: p=0.063<br>vs V3: 0.813	r=0.83<br>r=-0.11
HD/PD	IgG1 V3 (switched)	70.00 (32.15–70.55)	–	–
HD/PD	IgG1 V4 (switched)	70.20 (34.85–73.85)	–	–
HD/PD	IgG1 V3 (S-binding)	92.10 (52.10–93.55)	vs switched V3: p=0.125	r=0.68
HD/PD	IgG1 V4 (S-binding)	80.00 (42.15–89.50)	vs switched V4: p=0.625<br>vs V3: 0.813	r=0.22<br>r=-0.11
KTR	IgG1 V3 (switched)	43.50 (36.53–64.28)	–	–
KTR	IgG1 V4 (switched)	44.00 (36.65–69.65)	–	–
KTR	IgG1 V3 (S-binding)	77.65 (48.60–82.25)	vs switched V3: p=0.063	r=0.76

KTR	IgG1 V4 (S-binding)	81.20 (61.10–87.98)	vs switched V4: p=0.031<br>vs V3: 0.063	r=0.88<br>r=0.76
	Between groups IgG1 V3	–	p=0.518	ε²=0.10
	Between groups IgG1 V4	–	p=0.959	ε²=0.01

IgG2+

**Group**	**Timepoint / Population (%)**	**Median (IQR)**	**p**	**Effect size (r / ε²)**
CTRL	IgG2 V3 (switched)	22.55 (14.75–25.85)	–	–
CTRL	IgG2 V4 (switched)	22.30 (16.73–24.68)	–	–
CTRL	IgG2 V3 (S-binding)	4.55 (2.86–6.67)	vs switched V3: p=0.008	r=-0.94
CTRL	IgG2 V4 (S-binding)	0.67 (0.00–2.41)	vs switched V4: p=0.008<br>vs V3: 0.0312	r=-0.94<br>r=-0.88
CKD	IgG2 V3 (switched)	34.40 (19.60–38.35)	–	–
CKD	IgG2 V4 (switched)	30.80 (21.15–36.35)	–	–
CKD	IgG2 V3 (S-binding)	3.94 (0.00–6.85)	vs switched V3: p=0.063	r=-0.84
CKD	IgG2 V4 (S-binding)	1.45 (0.26–3.58)	vs switched V4: p=0.063<br>vs V3: 0.375	r=-0.84<br>r=-0.45
HD/PD	IgG2 V3 (switched)	23.00 (17.20–38.65)	–	–
HD/PD	IgG2 V4 (switched)	21.50 (14.60–36.20)	–	–
HD/PD	IgG2 V3 (S-binding)	5.26 (0.99–19.78)	vs switched V3: p=0.188	r=-0.59
HD/PD	IgG2 V4 (S-binding)	0.00 (0.00–7.68)	vs switched V4: p=0.063<br>vs V3: 0.875	r=-0.84<br>r=-0.08
KTR	IgG2 V3 (switched)	34.45 (16.60–51.08)	–	–
KTR	IgG2 V4 (switched)	34.80 (18.60–48.95)	–	–
KTR	IgG2 V3 (S-binding)	8.12 (2.83–29.05)	vs switched V3: p=0.031	r=-0.88
KTR	IgG2 V4 (S-binding)	14.35 (0.00–21.03)	vs switched V4: p=0.031<br>vs V3: 0.625	r=-0.88<br>r=0.22
	Between groups IgG2 V3	–	p=0.543	ε²=0.09
	Between groups IgG2 V4	–	p=0.442	ε²=0.12

IgG3+

**Group**	**Timepoint / Population (%)**	**Median (IQR)**	**p**	**Effect size (r / ε²)**
CTRL	IgG3 V3 (switched)	10.15 (6.58–13.58)	–	–
CTRL	IgG3 V4 (switched)	10.45 (6.39–12.45)	–	–
CTRL	IgG3 V3 (S-binding)	9.99 (5.28–12.35)	vs switched V3: p=0.742	r=-0.12
CTRL	IgG3 V4 (S-binding)	3.40 (1.70–9.32)	vs switched V4: p=0.055<br>vs V3: 0.016	r=-0.68<br>r=-0.86

CKD	IgG3 V3 (switched)	6.60 (5.70–7.60)	–	–
CKD	IgG3 V4 (switched)	7.46 (5.45–7.90)	–	–
CKD	IgG3 V3 (S-binding)	11.00 (7.60–22.40)	vs switched V3: p=0.125	r=0.68
CKD	IgG3 V4 (S-binding)	4.64 (3.46–8.88)	vs switched V4: p=0.438<br>vs V3: 0.188	r=-0.35<br>r=-0.59
HD/PD	IgG3 V3 (switched)	5.11 (2.69–21.74)	–	–
HD/PD	IgG3 V4 (switched)	4.51 (2.40–20.26)	–	–
HD/PD	IgG3 V3 (S-binding)	0.00 (0.00–32.64)	vs switched V3: p=0.813	r=-0.11
HD/PD	IgG3 V4 (S-binding)	1.02 (0.00–13.85)	vs switched V4: 0.438<br>vs V3: 0.750	r=-0.35<br>r=0.14
KTR	IgG3 V3 (switched)	8.41 (4.97–13.90)	–	–
KTR	IgG3 V4 (switched)	7.86 (5.16–14.08)	–	–
KTR	IgG3 V3 (S-binding)	10.39 (5.36–24.70)	vs switched V3: p=0.438	r=0.32
KTR	IgG3 V4 (S-binding)	4.78 (2.03–7.26)	vs switched V4: p=0.031<br>vs V3: 0.063	r=-0.88<br>r=-0.76
	Between groups IgG3 V3	–	p=0.394	ε^2=0.13
	Between groups IgG3 V4	–	p=0.781	ε^2=0.05

IgG4+

Group	Timepoint / Population (%)	Median (IQR)	p	Effect size (r / ε^2)
CTRL	IgG4 V3 (switched)	9.46 (5.50–16.23)	–	–
CTRL	IgG4 V4 (switched)	6.81 (6.27–12.70)	–	–
CTRL	IgG4 V3 (S-binding)	2.25 (2.24–5.04)	vs switched V3: p=0.055	r=-0.50
CTRL	IgG4 V4 (S-binding)	14.25 (6.25–24.30)	vs switched V4: p=0.148<br>vs V3: 0.016	r=0.55<br>r=0.86
CKD	IgG4 V3 (switched)	5.00 (2.76–10.80)	–	–
CKD	IgG4 V4 (switched)	4.48 (2.94–11.20)	–	–
CKD	IgG4 V3 (S-binding)	0.00 (0.00–0.90)	vs switched V3: p=0.063	r=-0.95
CKD	IgG4 V4 (S-binding)	16.10 (2.06–20.30)	vs switched V4: p=0.188<br>vs V3: 0.125	r=0.74<br>r=0.68
HD/PD	IgG4 V3 (switched)	4.51 (2.27–12.15)	–	–
HD/PD	IgG4 V4 (switched)	5.71 (3.19–12.30)	–	–
HD/PD	IgG4 V3 (S-binding)	0.00 (0.00–0.99)	vs switched V3: p=0.063	r=-0.89
HD/PD	IgG4 V4 (S-binding)	0.00 (0.00–56.30)	vs switched V4: p=0.813<br>vs V3: 0.500	r=-0.13<br>r=0.05
KTR	IgG4 V3 (switched)	4.77 (2.37–9.71)	–	–
KTR	IgG4 V4 (switched)	4.11 (3.14–5.80)	–	–
KTR	IgG4 V3 (S-binding)	0.00 (0.00–1.61)	vs switched V3: p=0.031	r=-0.60

KTR	IgG4 V4 (S-binding)	4.62 (0.00–14.33)	vs switched V4: p>0.999<br>vs V3: 0.188	r=0.01<br>r=0.59
	Between groups IgG4 V3	–	p=0.077	ε²=0.30
	Between groups IgG4 V4	–	p=0.437	ε²=0.12
